# The relationship between axial length, age and intraocular pressure in children with primary congenital glaucoma

**DOI:** 10.1038/s41598-020-74126-5

**Published:** 2020-10-20

**Authors:** Ibrahim Al-Obaida, Adi Mohammed Al Owaifeer, Khabir Ahmad, Rizwan Malik

**Affiliations:** 1 Glaucoma Division, King Khaled Eye Specialist Hospital, Riyadh, Saudi Arabia; 2 Faculty of Ophthalmology, College of Medicine, King Faisal University, Al-Hasa, Saudi Arabia; 3 Research Department, King Khaled Eye Specialist Hospital, AlArubah Branch Rd, Riyadh, 12329 Saudi Arabia

**Keywords:** Glaucoma, Ocular hypertension

## Abstract

Whilst axial length (AxL) from ultrasound examination is a useful clinical parameter for monitoring progression in younger children with glaucoma, distinguishing AxL changes due to raised intraocular pressure (IOP) from age is often challenging. Existing normograms have included a limited number of children with glaucoma. The aim of this study was to evaluate the relationship between AxL with age and IOP in children with primary congenital glaucoma (PCG) and develop a model for expected AxL increase with age. All children (n = 208; 397 eyes) with PCG who attended our tertiary eye care facility from June 2014 and July 2018 and had AxL and IOP measurements were included. The relationship of AxL with age and IOP was studied by applying a LOWESS fit and then mixed effects models. In the final model, age was the most significant factor influencing the growth of AxL (coefficient age 3.14[95% CI 2.91–3.35, *p* < 0.001], coefficient age^2^ − 0.53[95% CI, − 0.59 to − 0.47, *p* < 0.001]), and this association was influenced by the interaction of IOP with sex (*p* = 0.098 for girls relative to boys), the number of antiglaucoma medications (AGM [*p* < 0.001 for ≥ 3 AGM]) and glaucoma surgery (*p* = 0.015). This model enabled us to derive predicted values for clinical use in children with PCG to predict those with progressive glaucoma.

## Introduction

Primary congenital glaucoma (PCG) is a sporadic or inherited disease caused by an anomaly of the trabecular meshwork and anterior chamber angle causing an elevation of intraocular pressure. PCG is typically diagnosed within the first year of life^1^. Aside from elevated intraocular pressure (IOP), PCG is characterized by enlargement of the globe (buphthalmos), corneal edema, opacification of the cornea with rupture of Descemet's membrane (Haab’s striae) and progressive optic disc cupping^2^. The birth prevalence of PCG is around 1.5 per 100,000 live births in western countries^3^, but much commoner in some parts of the Middle East^4^, where it accounts for a substantial proportion of childhood blindness^5^.

Axial myopia is a common finding in PCG^6, 7^. As such, axial length (AxL), by ultrasound measurement, is one of the major investigative tools that plays a role in diagnosing and identifying progression in children with PCG^8–10^. Moreover, an increase in AxL is well-established in children with poorly controlled glaucoma^8, 10–12^.

Despite its usefulness, distinguishing AxL changes due to raised IOP *from* age is often challenging. During the first few years of life, the normal AxL growth follows a specific pattern^8^, being non-linear in nature with a steep increase in the first 2 years after birth and reaching a plateau after 3–4 years of age^10, 13, 14^. Childhood glaucoma is known to produce inflated AxL changes for age^10^, but there is a scarcity of data detailing the nature of the effect of IOP on AxL. Further, individuals with PCG have thinner sclera^15^, with likely different physical properties to healthy children. As such, normograms for AxL change in healthy children^13, 16^ may not be applicable to children with PCG.

The aim of this study was to evaluate the relationship between AxL with age and IOP in children with an established diagnosis of PCG and develop a normogram for expected AxL increase with age for this group of children.

## Methods

### Study design

This was a retrospective cohort study, with data on AxL and IOP collected for all eligible children who attended our tertiary eye care centre since the initiation of our electronic patient record system (TrakCare, Intersystems, Cambridge, MA). Children were typically followed up after diagnosis in the Pediatric or Glaucoma Division every two to six months, depending on their age, IOP and degree of optic disc damage.

The records of patients with a diagnosis of PCG who attended our facility between June 2014 and July 2018 in King Khaled Eye Specialist Hospital (KKESH) with a diagnosis of ‘pediatric glaucoma’ (by hospital code) were reviewed. The study protocol was approved by the Institutional Review Board (IRB) of KKESH (IRB no 1837-R). The study was carried out in accordance with institutional ethics guidelines. The need for informed consent was waived by the IRB of KKESH because of the retrospective nature of the study. The study adhered to the tenets of the Declaration of Helsinki.

### Inclusion and exclusion criteria

All children ≤ 4 years of age who attended during the study period, were included. In case of unilateral disease only affected eye were included. For children with bilateral PCG, which accounted for the majority of children, both eyes were included. The analysis accounted for inclusion of both eyes from each child (see ‘*data analysis*’ below) to account for inter-eye correlation. Eyes with secondary glaucoma, blind or phthisical eyes or with no axial length data during the study period were excluded.

### Diagnostic criteria

For the purpose of this study, our definition of PCG was consistent with that given by the World Glaucoma Consensus Committee^17^. PCG consisted of childhood glaucoma, in the absence of a secondary cause, which presented in the first month of birth with characteristic clinical features including corneal signs (buphthalmos, Haab striae, corneal clouding), raised IOP, optic disc cupping and axial myopia. It encompassed neonatal and infantile subtypes^17^.

### IOP and AxL measurement

All IOP and AxL measurements were taken either under after sedation or prior to anesthesia before intubation. IOP was measured using Tonopen AVIA (TPA, Reichert Inc. Buffalo, NY) or the Reichert Model 30 pneumatonometer (Reichert Inc. Buffalo, NY). Children were examined under inhalation anesthesia before intubation or under sedation as follow-up visit (using oral chloral hydrate, 100 mg/ml solution, which has no appreciable effect on IOP measurements)^18^. Axial length measurements were taken by an experienced ophthalmic technician utilizing a 10 MHz A-scan machine (Cinescan, Quantel Medical, Cournon d'Auvergne, France) using contact biometry. After application of a topical anesthetic agent (0.4% Oxybuprocaine hydrochloride), the probe was placed gently on the patient's cornea. Characteristic waves representing cornea, lens, retina, and sclera were observed. Prior to taking a reading the ultrasound waves were evaluated an in cases of noisy peaks, the reading was repeated. The axial length was defined as the distance from the wave representing the anterior corneal surface to the wave representing the retina at the macula. In each patient, the machine recorded five accurate axial length measurements and gave an average reading of all measurements.

### Data collection

The following baseline data were collected: age, gender, IOP, ocular axial length, number and type of any previous ocular surgeries, and number anti-glaucoma medications (AGM). For each follow-up visit, the age of the child, IOP, AxL, AGM, clinical status and the need for surgery was recorded.

### Data analysis

Data were entered using Microsoft Excel 2010 (Microsoft Corporation, Redmond, Washington) and analyzed using STATA 16.0 (StataCorp LLC, College Station, TX, USA). For the description of baseline and other characteristics, categorical data were presented as frequencies and percentages / continuous data were presented as means with standard deviation or medians with interquartile range, depending on the normality of the data.

The AxL versus time data were analyzed in four stages. The first stage involved an exploratory data analysis to visualize trends in the data. A scatterplot of AxL (mm) versus age (years) was plotted for all children at 2 different IOP cut-offs: those with IOP ≤ 21 mmHg and those with IOP > 21 mmHg on any visit. This IOP cut-off was chosen as it has typically been used to classify abnormally high IOP in glaucoma studies involving children^1^.

Secondly, to predict trends in the data, a locally weighted smoothing (LOWESS)^19^ curve was fitted to estimate the relationship of AxL and age for the two groups. A LOWESS curve was used for initial fitting as this method does not make assumptions about the type of fit to the data.

Lastly, the effect of age and level of IOP on AxL was estimated using linear mixed model regression analysis, for all included eyes, using: age, gender, IOP, AGM and ‘surgery needed’ as explanatory variables and using patient ID and eye as random intercepts to account for multiple observations per patient and per eye. Four mixed effect models were developed. For determining the inclusion of a factor which improved the fit, − 2 Log likelihood and Akaike's information criterion (AIC) values were used. The model with the lowest − 2 Log Likelihood and AIC values was considered to have the best fit. The first model (*Model 1*) included linear and quadratic terms of age to better capture the time-changing shape of the axial length growth curve while the second model, in addition, included IOP. The third model added sex. The full model (*Model 4*) which additionally contained IOP-sex interaction term, AGM and surgery needed during the follow up, had the lowest − 2 Log Likelihood and AIC values and was thus used for inference.

STATA's *margins* command was used to calculate gender-specific predicted AxL for different age and IOP levels (Supplement Table 1). The command is used to compute predictions from a regression model.

Subsequently, *marginsplot* command was used to graph predicted axial length from fitted model for IOPs of 12, 15, 18 and 21, 25 and 30 mmHg for both boys and girls from birth up to 3 years of age.

## Results

The dataset included 397 eyes of 208 children (105 boys and 103 girls). The mean (± SD) age of children at the first visit was 0.92 ± 1.03 years (Minimum = 2 days, maximum = 3.99 years) (Table 1). Of 397 eyes, follow-up data were available for a mean period of 1.02 ± 0.95 years. Overall, 278 (70%) eyes had ≤ 3 visits and 119(30%) eyes had more than 3 visits.

**Table 1:** Baseline characteristics of study population (397 eyes of 208 children) at the first visit.

Parameter	Value
Age (years), mean ± SD	0.92 ± 1.03
Gender (boys: girls), number of eyes	197: 200
Number of anti-glaucoma medications, mean ± SD	1.92 ± 1.54
IOP (mmHg), mean ± SD	23.99 ± 8.78
Axial length (mm), mean ± SD	22.32 ± 2.60
Previous glaucoma surgery, number of eyes (%)	163 (41.1%)

SD, standard deviation; IOP, intraocular pressure.

**Figure 1:**
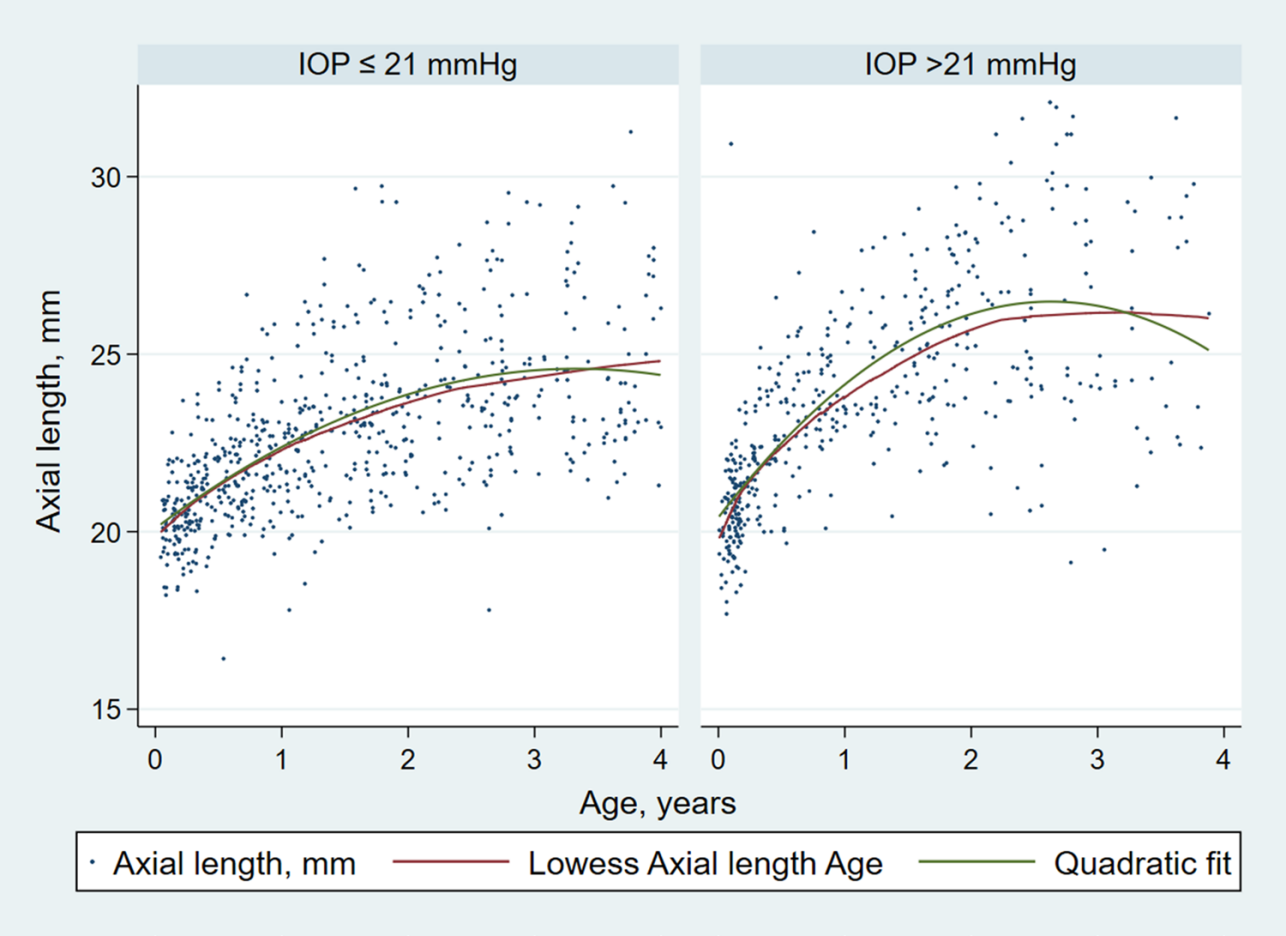
Scatterplot of whole data for children with (**a**) normal IOP (≤ 21 mmHg) and (**b**) children with IOP > 21 mmHg at any visit. LOWESS and quadratic fits to the data are shown.

The raw AxL data plotted against age, with the LOWESS and quadratic fits are shown in Fig. 1. As expected, there was a non-linear relationship between AxL and age, irrespective of whether the IOP was ≤ 21 mmHg or IOP > 21 mmHg on any visit. However, the curve was steeper all the corresponding time points for IOP > 21 mmHg.

Table 2 shows 4 linear mixed models of the relationship between axial length and age, with and without IOP, sex and treatment as affecting factors. As expected, age was the most significant factor influencing the growth of axial length. Age and its quadratic term were found to show significant and consistent relationship with the AxL. However, this association was significantly influenced by IOP, sex, the number of glaucoma medications and the decision for glaucoma surgery. The final model (*Model 4*), comprising of age, IOP, sex, IOP*sex, AGM and Surgery needed, had the lowest − 2 log likelihood and AIC values.

**Table 2:** Linear mixed models regression analysis of the relationship between axial length and age, with and without IOP, sex and treatment as affecting factors.

Axial length	Model 1		Model 2		Model 3		Model 4	
	Coeff. (95% CI)	*P*	Coeff. (95% CI)	*P*	Coeff. (95% CI)	*P*	Coeff. (95% CI)	*P*
Age	2.78(2.56, 3.00)	< 0.001	3.05(2.84, 3.27)	< 0.001	3.05(2.83, 3.27)	< 0.001	3.14(2.91, 3.35)	< 0.001
Age^2^	− 0.47(− 0.53, − 0.41)	< 0.001	− 0.51(− 0.57, − 0.45)	< 0.001	− 0.51(− 0.57, − 0.45)	< 0.001	− 0.53 (− 0.59, − 0.47)	< 0.001
IOP			0.05 (0.04, 0.06)	< 0.001	0.05 (0.04, 0.06)	< 0.001	0.05 (0.04, 0.06)	< 0.001
**Sex**								
Female					− 0.52 (− 0.97, − 0.06)	0.026	− 0.22 (− 0.78, 0.34)	0.449
IOP*Sex								
Female							− 0.01 (− 0.03, 0.002)	0.098
**AGM**								
None							Reference	
1–2							0.18 (− 0.01, 0.37)	0.058
≥ 3							0.40 (0.22, 0.57)	< 0.001
**Surgery needed**								
Yes							0.22(0.04, 0.39)	0.015
No							Reference	
− 2 log likelihood	3979.1862		3852.238		3847.3162		3807.4364	
AIC value	3991.186		3866.239		3863.316		3831.436	

IOP, intraocular pressure; AGM, antiglaucoma medication; AIC, Akaike information criterion.

**Figure 2:**
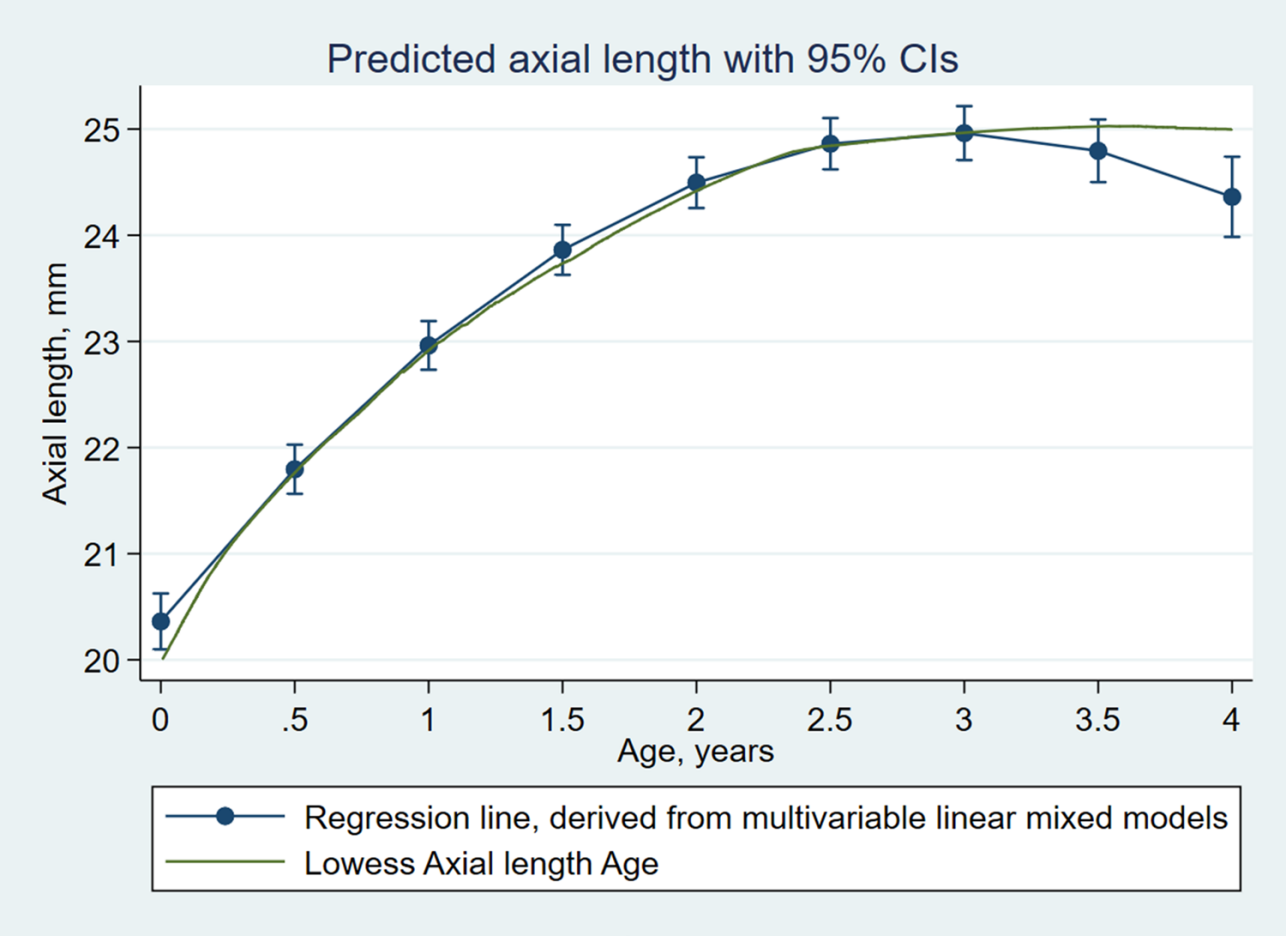
Linear mixed models based plot of predicted axial length for different age and different levels of IOP, with superimposed LOWESS curve.

**Figure 3:**
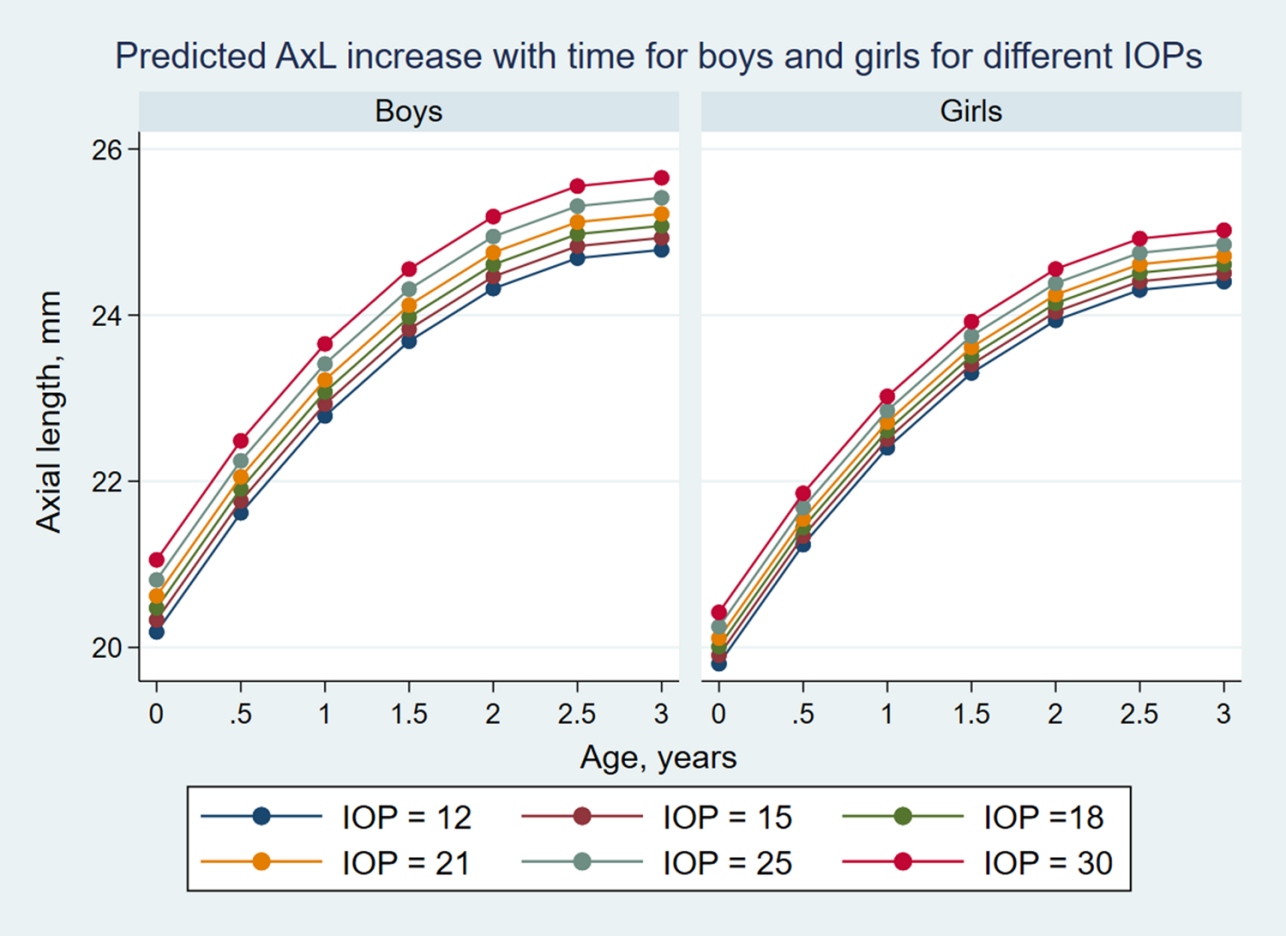
Predicted axial length increase with age for boys and girls, for different levels of IOP.

**Figure 4:**
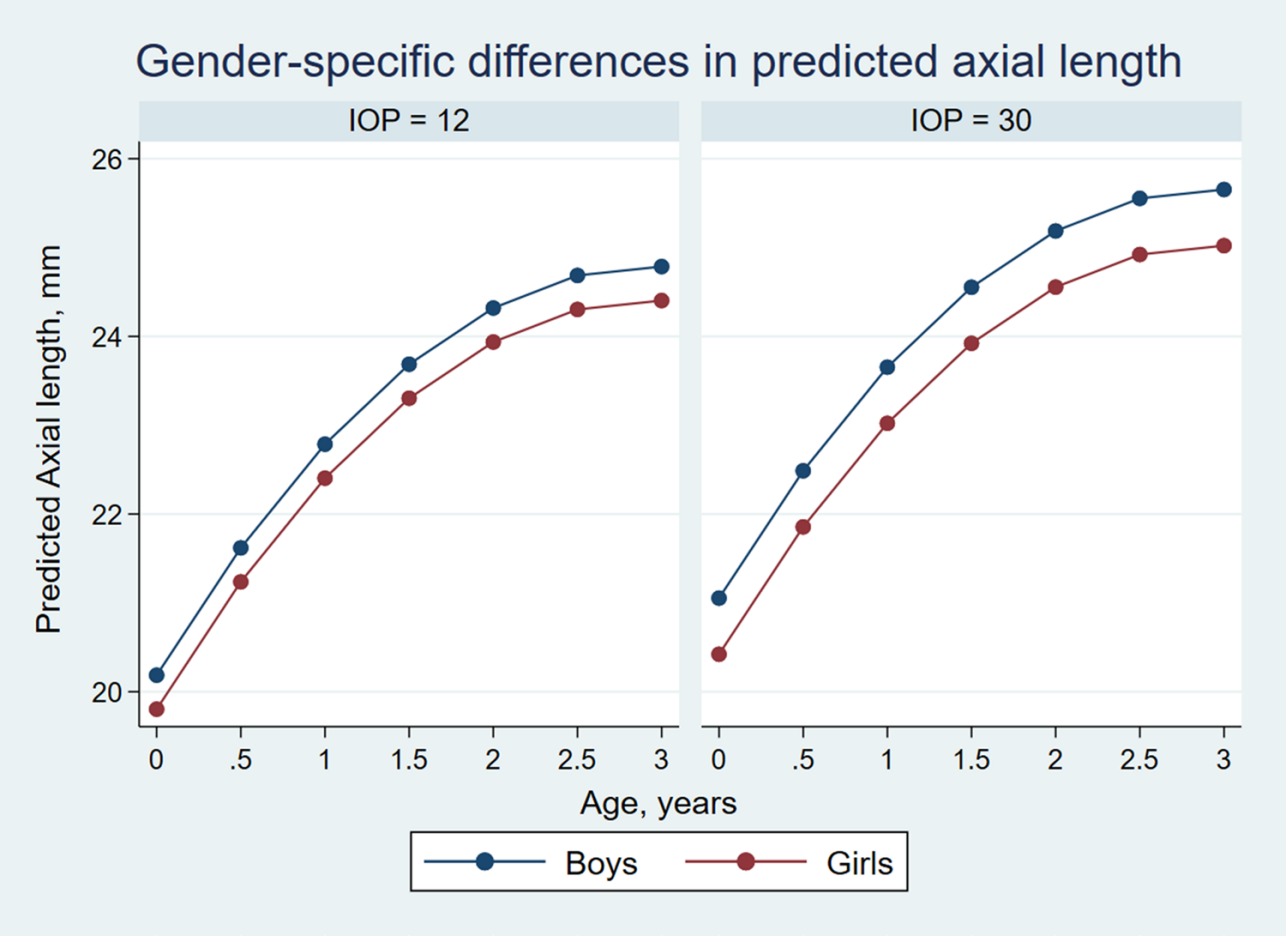
Gender-specific differences in predicted axial length with age for IOPs 12 mmHg and 30 mmHg.

The curve derived from the final model approximated to the LOWESS closely (Fig. 2). As AxL increase differed between sexes, further trends were described for boys and girls separately. Figure 3 shows the predicted AxL increase from the model for boys and girls with time for IOPs of 12, 15, 18, 21, 25 and 30 mmHg. The figure shows that overall boys tended to have greater AxL growth compared to girls for different levels of IOP. The interaction between IOP and sex reached marginal significance. The maxima of the curves were at around 3 years for both the sexes. Further, the changes in AxL between the boys and the girls were visibly more pronounced for a higher IOP than normal and near normal values (Fig. 4).

**Figure 5:**
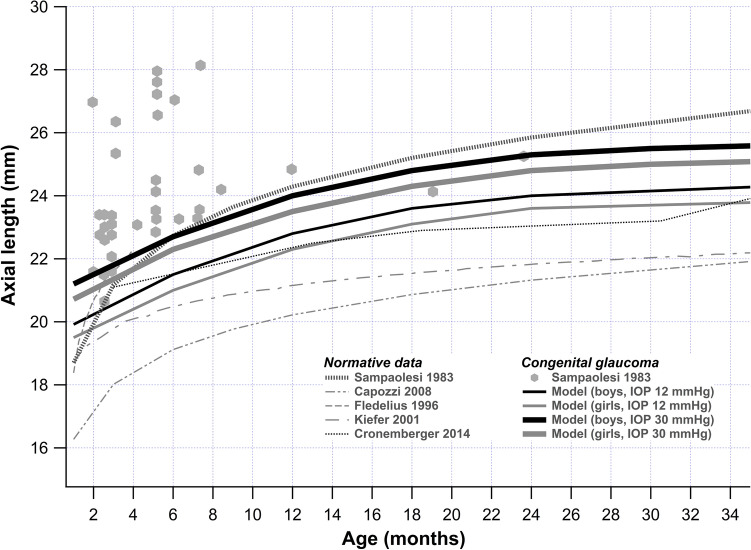
Comparison of model prediction of our study with other studies from the literature.

Figure 5 shows the results of our model for IOPs of 12 and 30 mmHg (for boys and girls) compared with other studies from the literature.

## Discussion

The purpose of this study was to establish the relationship between axial length, age and IOP in a cohort of children with PCG. We found a curvilinear relationship between AxL and age which was strongly influenced by IOP and which reached a plateau after around 3 years of age. Further, we found gender differences for this relationship, with boys tending to have a slightly greater AxL for a given age than girls.

AxL measurement is an indispensable tool in young children with reasonable repeatability^20^. There are limited tools to predict progressive disease in younger children. While older children can often perform kinetic perimetry with acceptable reliability^21–23^, assessment of glaucomatous progression in younger children is often challenging and relies mainly on objective clinical measures. These include corneal diameter, axial length and cup-disc-ratio. The latter is sometimes not possible in children with corneal opacification. In order to identify progressive disease, clinicians need to know the normal range of AxL for children of a given age. The results of this study enabled derivation of a normogram, providing estimates of 95% confidence intervals of children with PCG, which can be used for monitoring AxL in children with PCG in the clinical setting.

In our study, we found that AxL reaches a plateau around 3 years in both boys and girls. AxL measurement beyond this age may, therefore, not be clinically useful. Although other studies have not specifically examined the age at which AxL increase appears to cease, data from other studies seems consistent with our study. For example, data from eight healthy eyes in Kiefer’s^9^ and thirty-nine healthy eyes from Capozzi’s study^13^, seems to show a flattening of AxL increase after 3 years of age. Our findings seem physiologically plausible, as the sclera becomes much less distensible after 3 years of age^24^.

Like us, others have also reported a non-linear relationship of AxL with age in children^9, 10, 13^, with data that was broadly consistent with our model (Fig. 5). While others have tended to fit a log function to the data^10^, we found that the addition of a quadratic term for age help capture AxL growth curve better. With increasing IOP, the curve was displaced upwards, giving a higher AxL for the same age.

A novel finding of our study was the gender-dependence of the AxL increase with age. Boys have a slightly higher AxL at all ages (for a given level of IOP) than girls^25^. Adult men have, on average, greater AxLs than women^26, 27^. Also, as a positive correlation exists between axial length and height in children^26^, it follows that boys would have a longer eye than girls of the same age. In one study of children with retinopathy of prematurity, Laws et al. noted that boys had an axial length which was consistently longer, by an average of 0.30 mm compared to girls^28^.

The predicted association of AxL with IOP is subject to numerous sources of error: most notably, the test–retest variability of IOP and AxL measurements. Tonopen and pneumotonometry measurements have been generally found to be reliable in children, exhibiting small differences compared to Goldmann applanation tonometry^29^. In our study, we averaged IOP measurements taken on the same visit by different instruments—so that an average of 2 or more measurements at the same visit were used in over 90% of visits. This is likely to significantly reduce the variability of single IOP measurements.

Studies on the reproducibility of AxL measurements relate mostly to adult eyes^30, 31^. These show that axial length measurements are highly reproducible, with a coefficient of variation of less than 1%^30^.

Two major strengths of the current study are the large dataset which included nearly 400 eyes with serial IOP and AxL measurements and a relatively large amount of data on a condition which, itself is relatively rare. This is a significant addition to the current literature. The largest study of children with PCG comes included 144 eyes^4^. Secondly, all IOP measurements in our study were undertaken with children, under 4 years old, who were either sedated or anaesthetized and so artefacts due to squeezing and movement have been largely avoided. Our data enabled us to derive a normogram for clinical use, which has direct practical application for monitoring children with PCG.

One of the limitations of the current study is the varying intervals and length of follow-up across children as the data was acquired as part of routine clinical practice and not specifically for this study. A prospective study that utilizes a more controlled and uniform environment for the measurement of IOP and AxL would also be useful. This should include multiple IOP and AxL measurements per visit at fixed longitudinal intervals and masking of the ultrasonographer to the clinical characteristics of the children, including IOP. A mixed-effects method was used to account for varying lengths of follow-up, correlation between the two eyes of the same child and for autocorrelation of repeated measurements over time. Additionally, analytic approaches such as quantile regression^32^ that are distribution agnostic can be used to examine the relationship between AxL and predictors (such as age, IOP, gender, AGM, and surgery in the same patient population) to provide more reliable estimates of AxL confidence intervals.

There were two reasons for not including a control group of children without glaucoma in the current study: firstly, being a tertiary care hospital, our facility has very few healthy children who attend routinely for ophthalmic examination. Examining such children under anaesthesia or sedation poses feasibility and ethical issues; secondly, there are already ample studies in the literature which have examined the relationship between AxL and age in healthy children^7, 9, 13^.

In the present study, we did not examine the relationship between different measures of glaucomatous progression in children. Apart from axial length, there are other markers of glaucomatous progression in young children, including corneal diameter and cup-disc-ratio. Many of our children with PCG have a degree of corneal opacification, making assessment of the cup-disc-ratio difficult.

In conclusion, we identified a curvilinear relationship between AxL and age in young children which was highly dependent on IOP and gender.

Further work is needed to validate the predicted model in different cohorts of children and to evaluate its applicability in children of Caucasian and other races. Studies are also needed to further investigate the hypothesis that girls may be more resistant to AxL increase compared to boys at similar levels of IOP. An interactive web-based tool^33^ to allow longitudinal against predicted AxL measurements would be a useful adjunct to this study.

## Supplementary information

Supplementary Table.
