# Supplementary material for: The relationship between axial length, age and intraocular pressure in children with primary congenital glaucoma
Source: Sci Rep. 2020 Oct 20;10:17821. doi: 10.1038/s41598-020-74126-5 (PMC7575558; doi:10.1038/s41598-020-74126-5)
Supplement: Supplementary file 1 — Supplementary Table. [file 41598_2020_74126_MOESM1_ESM.pdf]

S1 -Predicted values of AxL (mm) for different ages

		At birth			6 months			12 months			18 months			24 months			30 months			36 months		
**IOP**	**Sex**	Axl	LCI	UCI	Axl	LCI	UCI	Axl	LCI	UCI	Axl	LCI	UCI	Axl	LCI	UCI	Axl	LCI	UCI	Axl	LCI	UCI
**5**	*Boy*	19.8	19.4	20.3	21.3	20.9	21.7	22.4	22.1	22.8	23.3	23.0	23.7	24.0	23.6	24.4	24.3	24.0	24.7	24.4	24.1	24.8
**5**	*Girl*	19.6	19.2	20.0	21.0	20.6	21.4	22.2	21.8	22.5	23.1	22.7	23.4	23.7	23.3	24.1	24.1	23.7	24.4	24.2	23.8	24.5
**6**	*Boy*	19.9	19.5	20.3	21.3	20.9	21.7	22.5	22.1	22.9	23.4	23.0	23.8	24.0	23.6	24.4	24.4	24.0	24.8	24.5	24.1	24.9
**6**	*Girl*	19.6	19.2	20.0	21.0	20.7	21.4	22.2	21.8	22.6	23.1	22.7	23.5	23.7	23.4	24.1	24.1	23.7	24.5	24.2	23.8	24.6
**7**	*Boy*	19.9	19.5	20.3	21.4	21.0	21.8	22.5	22.2	22.9	23.4	23.1	23.8	24.1	23.7	24.5	24.4	24.1	24.8	24.5	24.2	24.9
**7**	*Girl*	19.6	19.2	20.0	21.1	20.7	21.4	22.2	21.9	22.6	23.1	22.8	23.5	23.8	23.4	24.1	24.1	23.8	24.5	24.2	23.9	24.6
**8**	*Boy*	20.0	19.6	20.4	21.4	21.1	21.8	22.6	22.2	23.0	23.5	23.1	23.9	24.1	23.8	24.5	24.5	24.1	24.9	24.6	24.2	25.0
**8**	*Girl*	19.7	19.3	20.0	21.1	20.7	21.5	22.3	21.9	22.6	23.2	22.8	23.5	23.8	23.4	24.2	24.2	23.8	24.5	24.3	23.9	24.6
**9**	*Boy*	20.0	19.7	20.4	21.5	21.1	21.8	22.6	22.3	23.0	23.5	23.2	23.9	24.2	23.8	24.5	24.5	24.2	24.9	24.6	24.3	25.0
**9**	*Girl*	19.7	19.3	20.1	21.1	20.8	21.5	22.3	22.0	22.6	23.2	22.9	23.5	23.8	23.5	24.2	24.2	23.9	24.5	24.3	23.9	24.7
**10**	*Boy*	20.1	19.7	20.5	21.5	21.2	21.9	22.7	22.3	23.0	23.6	23.2	23.9	24.2	23.9	24.6	24.6	24.2	24.9	24.7	24.3	25.0
**10**	*Girl*	19.7	19.4	20.1	21.2	20.8	21.5	22.3	22.0	22.7	23.2	22.9	23.6	23.9	23.5	24.2	24.2	23.9	24.6	24.3	24.0	24.7
**11**	*Boy*	20.1	19.8	20.5	21.6	21.2	21.9	22.7	22.4	23.1	23.6	23.3	24.0	24.3	23.9	24.6	24.6	24.3	25.0	24.7	24.4	25.1
**11**	*Girl*	19.8	19.4	20.1	21.2	20.9	21.5	22.4	22.0	22.7	23.3	22.9	23.6	23.9	23.6	24.2	24.3	23.9	24.6	24.4	24.0	24.7
**12**	*Boy*	20.2	19.8	20.6	21.6	21.3	22.0	22.8	22.4	23.1	23.7	23.3	24.0	24.3	24.0	24.7	24.7	24.3	25.0	24.8	24.4	25.1
**12**	*Girl*	19.8	19.4	20.2	21.2	20.9	21.6	22.4	22.1	22.7	23.3	23.0	23.6	23.9	23.6	24.3	24.3	24.0	24.6	24.4	24.1	24.8
**13**	*Boy*	20.2	19.9	20.6	21.7	21.3	22.0	22.8	22.5	23.2	23.7	23.4	24.1	24.4	24.0	24.7	24.7	24.4	25.1	24.8	24.5	25.2
**13**	*Girl*	19.8	19.5	20.2	21.3	20.9	21.6	22.4	22.1	22.8	23.3	23.0	23.7	24.0	23.6	24.3	24.3	24.0	24.7	24.4	24.1	24.8
**14**	*Boy*	20.3	19.9	20.6	21.7	21.4	22.1	22.9	22.5	23.2	23.8	23.4	24.1	24.4	24.1	24.8	24.8	24.4	25.1	24.9	24.5	25.2
**14**	*Girl*	19.9	19.5	20.2	21.3	21.0	21.6	22.5	22.1	22.8	23.4	23.0	23.7	24.0	23.7	24.3	24.4	24.0	24.7	24.5	24.1	24.8
**15**	*Boy*	20.3	20.0	20.7	21.8	21.4	22.1	22.9	22.6	23.3	23.8	23.5	24.2	24.5	24.1	24.8	24.8	24.5	25.2	24.9	24.6	25.3
**15**	*Girl*	19.9	19.6	20.3	21.3	21.0	21.7	22.5	22.2	22.8	23.4	23.1	23.7	24.0	23.7	24.4	24.4	24.1	24.7	24.5	24.2	24.8
**16**	*Boy*	20.4	20.0	20.7	21.8	21.5	22.1	23.0	22.7	23.3	23.9	23.5	24.2	24.5	24.2	24.8	24.9	24.6	25.2	25.0	24.6	25.3
**16**	*Girl*	19.9	19.6	20.3	21.4	21.1	21.7	22.5	22.2	22.9	23.4	23.1	23.8	24.1	23.7	24.4	24.4	24.1	24.8	24.5	24.2	24.9
**17**	*Boy*	20.4	20.1	20.8	21.9	21.5	22.2	23.0	22.7	23.4	23.9	23.6	24.3	24.6	24.2	24.9	24.9	24.6	25.3	25.0	24.7	25.4
**17**	*Girl*	20.0	19.6	20.3	21.4	21.1	21.7	22.6	22.3	22.9	23.5	23.2	23.8	24.1	23.8	24.4	24.5	24.1	24.8	24.6	24.2	24.9
**18**	*Boy*	20.5	20.1	20.8	21.9	21.6	22.2	23.1	22.8	23.4	24.0	23.7	24.3	24.6	24.3	24.9	25.0	24.7	25.3	25.1	24.7	25.4
**18**	*Girl*	20.0	19.7	20.4	21.4	21.1	21.8	22.6	22.3	22.9	23.5	23.2	23.8	24.1	23.8	24.5	24.5	24.2	24.8	24.6	24.3	24.9
**19**	*Boy*	20.5	20.2	20.9	22.0	21.6	22.3	23.1	22.8	23.4	24.0	23.7	24.3	24.7	24.3	25.0	25.0	24.7	25.3	25.1	24.8	25.5
**19**	*Girl*	20.0	19.7	20.4	21.5	21.2	21.8	22.6	22.3	23.0	23.5	23.2	23.9	24.2	23.9	24.5	24.5	24.2	24.9	24.6	24.3	25.0
**20**	*Boy*	20.6	20.2	20.9	22.0	21.7	22.3	23.2	22.9	23.5	24.1	23.7	24.4	24.7	24.4	25.0	25.1	24.7	25.4	25.2	24.8	25.5
**20**	*Girl*	20.1	19.7	20.4	21.5	21.2	21.8	22.7	22.4	23.0	23.6	23.3	23.9	24.2	23.9	24.5	24.6	24.2	24.9	24.7	24.3	25.0
**21**	*Boy*	20.6	20.3	21.0	22.1	21.7	22.4	23.2	22.9	23.5	24.1	23.8	24.4	24.8	24.4	25.1	25.1	24.8	25.4	25.2	24.9	25.6
**21**	*Girl*	20.1	19.8	20.5	21.5	21.2	21.9	22.7	22.4	23.0	23.6	23.3	23.9	24.2	23.9	24.6	24.6	24.3	24.9	24.7	24.4	25.1
**22**	*Boy*	20.7	20.3	21.0	22.1	21.8	22.4	23.3	22.9	23.6	24.2	23.8	24.5	24.8	24.5	25.1	25.2	24.8	25.5	25.3	24.9	25.6
**22**	*Girl*	20.1	19.8	20.5	21.6	21.3	21.9	22.7	22.4	23.1	23.6	23.3	24.0	24.3	24.0	24.6	24.6	24.3	25.0	24.7	24.4	25.1
**23**	*Boy*	20.7	20.4	21.1	22.1	21.8	22.5	23.3	23.0	23.6	24.2	23.9	24.5	24.8	24.5	25.2	25.2	24.9	25.5	25.3	25.0	25.6
**23**	*Girl*	20.2	19.8	20.5	21.6	21.3	21.9	22.8	22.5	23.1	23.7	23.4	24.0	24.3	24.0	24.6	24.7	24.4	25.0	24.8	24.4	25.1
**24**	*Boy*	20.8	20.4	21.1	22.2	21.9	22.5	23.4	23.0	23.7	24.3	23.9	24.6	24.9	24.6	25.2	25.3	24.9	25.6	25.4	25.0	25.7
**24**	*Girl*	20.2	19.9	20.6	21.6	21.3	22.0	22.8	22.5	23.1	23.7	23.4	24.0	24.3	24.0	24.7	24.7	24.4	25.0	24.8	24.5	25.2
**25**	*Boy*	20.8	20.5	21.2	22.2	21.9	22.6	23.4	23.1	23.7	24.3	24.0	24.6	24.9	24.6	25.3	25.3	25.0	25.6	25.4	25.1	25.7
**25**	*Girl*	20.3	19.9	20.6	21.7	21.4	22.0	22.9	22.5	23.2	23.8	23.4	24.1	24.4	24.1	24.7	24.8	24.4	25.1	24.9	24.5	25.2
**26**	*Boy*	20.9	20.5	21.2	22.3	22.0	22.6	23.5	23.1	23.8	24.4	24.0	24.7	25.0	24.7	25.3	25.4	25.0	25.7	25.5	25.1	25.8
**26**	*Girl*	20.3	19.9	20.6	21.7	21.4	22.0	22.9	22.6	23.2	23.8	23.5	24.1	24.4	24.1	24.8	24.8	24.4	25.1	24.9	24.5	25.2
**27**	*Boy*	20.9	20.6	21.3	22.3	22.0	22.7	23.5	23.2	23.8	24.4	24.1	24.7	25.0	24.7	25.4	25.4	25.1	25.7	25.5	25.2	25.9
**27**	*Girl*	20.3	20.0	20.7	21.8	21.4	22.1	22.9	22.6	23.2	23.8	23.5	24.1	24.5	24.1	24.8	24.8	24.5	25.2	24.9	24.6	25.3

**28**	*Boy*	21.0	20.6	21.3	22.4	22.1	22.7	23.6	23.2	23.9	24.5	24.1	24.8	25.1	24.8	25.4	25.5	25.1	25.8	25.6	25.2	25.9
**28**	*Girl*	20.4	20.0	20.7	21.8	21.5	22.1	23.0	22.6	23.3	23.9	23.5	24.2	24.5	24.1	24.8	24.9	24.5	25.2	25.0	24.6	25.3
**29**	*Boy*	21.0	20.6	21.4	22.4	22.1	22.8	23.6	23.3	23.9	24.5	24.2	24.8	25.1	24.8	25.5	25.5	25.2	25.8	25.6	25.3	26.0
**29**	*Girl*	20.4	20.0	20.7	21.8	21.5	22.1	23.0	22.7	23.3	23.9	23.6	24.2	24.5	24.2	24.9	24.9	24.5	25.2	25.0	24.6	25.3
**30**	*Boy*	21.1	20.7	21.4	22.5	22.1	22.8	23.7	23.3	24.0	24.6	24.2	24.9	25.2	24.8	25.5	25.6	25.2	25.9	25.7	25.3	26.0
**30**	*Girl*	20.4	20.1	20.8	21.9	21.5	22.2	23.0	22.7	23.4	23.9	23.6	24.3	24.6	24.2	24.9	24.9	24.6	25.3	25.0	24.7	25.4
**31**	*Boy*	21.1	20.7	21.5	22.5	22.2	22.9	23.7	23.4	24.0	24.6	24.3	24.9	25.2	24.9	25.6	25.6	25.3	26.0	25.7	25.3	26.1
**31**	*Girl*	20.5	20.1	20.8	21.9	21.6	22.2	23.1	22.7	23.4	24.0	23.6	24.3	24.6	24.2	24.9	25.0	24.6	25.3	25.1	24.7	25.4
**32**	*Boy*	21.2	20.8	21.5	22.6	22.2	22.9	23.8	23.4	24.1	24.7	24.3	25.0	25.3	24.9	25.6	25.6	25.3	26.0	25.7	25.4	26.1
**32**	*Girl*	20.5	20.1	20.8	21.9	21.6	22.3	23.1	22.8	23.4	24.0	23.6	24.3	24.6	24.3	25.0	25.0	24.6	25.3	25.1	24.7	25.5
**33**	*Boy*	21.2	20.8	21.6	22.6	22.3	23.0	23.8	23.4	24.2	24.7	24.3	25.1	25.3	25.0	25.7	25.7	25.3	26.1	25.8	25.4	26.2
**33**	*Girl*	20.5	20.2	20.9	22.0	21.6	22.3	23.1	22.8	23.5	24.0	23.7	24.4	24.7	24.3	25.0	25.0	24.7	25.4	25.1	24.7	25.5
**34**	*Boy*	21.2	20.9	21.6	22.7	22.3	23.0	23.8	23.5	24.2	24.7	24.4	25.1	25.4	25.0	25.7	25.7	25.4	26.1	25.8	25.5	26.2
**34**	*Girl*	20.6	20.2	20.9	22.0	21.6	22.3	23.2	22.8	23.5	24.1	23.7	24.4	24.7	24.3	25.1	25.1	24.7	25.4	25.2	24.8	25.5
**35**	*Boy*	21.3	20.9	21.7	22.7	22.4	23.1	23.9	23.5	24.3	24.8	24.4	25.2	25.4	25.1	25.8	25.8	25.4	26.2	25.9	25.5	26.3
**35**	*Girl*	20.6	20.2	21.0	22.0	21.7	22.4	23.2	22.8	23.5	24.1	23.7	24.5	24.7	24.4	25.1	25.1	24.7	25.5	25.2	24.8	25.6
**36**	*Boy*	21.3	20.9	21.7	22.8	22.4	23.2	23.9	23.6	24.3	24.8	24.5	25.2	25.5	25.1	25.9	25.8	25.5	26.2	25.9	25.6	26.3
**36**	*Girl*	20.6	20.3	21.0	22.1	21.7	22.4	23.2	22.9	23.6	24.1	23.8	24.5	24.8	24.4	25.1	25.1	24.7	25.5	25.2	24.8	25.6
**37**	*Boy*	21.4	21.0	21.8	22.8	22.4	23.2	24.0	23.6	24.4	24.9	24.5	25.3	25.5	25.1	25.9	25.9	25.5	26.3	26.0	25.6	26.4
**37**	*Girl*	20.7	20.3	21.0	22.1	21.7	22.5	23.3	22.9	23.6	24.2	23.8	24.5	24.8	24.4	25.2	25.2	24.8	25.5	25.3	24.9	25.7
**38**	*Boy*	21.4	21.0	21.8	22.9	22.5	23.3	24.0	23.7	24.4	24.9	24.5	25.3	25.6	25.2	26.0	25.9	25.5	26.3	26.0	25.6	26.4
**38**	*Girl*	20.7	20.3	21.1	22.1	21.8	22.5	23.3	22.9	23.7	24.2	23.8	24.6	24.8	24.4	25.2	25.2	24.8	25.6	25.3	24.9	25.7
**39**	*Boy*	21.5	21.1	21.9	22.9	22.5	23.3	24.1	23.7	24.5	25.0	24.6	25.4	25.6	25.2	26.0	26.0	25.6	26.4	26.1	25.7	26.5
**39**	*Girl*	20.7	20.3	21.1	22.2	21.8	22.5	23.3	23.0	23.7	24.2	23.8	24.6	24.9	24.5	25.3	25.2	24.8	25.6	25.3	24.9	25.7
**40**	*Boy*	21.5	21.1	22.0	23.0	22.6	23.4	24.1	23.7	24.5	25.0	24.6	25.4	25.7	25.3	26.1	26.0	25.6	26.5	26.1	25.7	26.6
**40**	*Girl*	20.8	20.4	21.2	22.2	21.8	22.6	23.4	23.0	23.7	24.3	23.9	24.7	24.9	24.5	25.3	25.3	24.9	25.7	25.4	24.9	25.8
